# Impact of sequelae of visceral leishmaniasis and their contribution to ongoing transmission of *Leishmania donovani*

**DOI:** 10.1093/femspd/ftz057

**Published:** 2019-10-07

**Authors:** Malcolm S Duthie, Yasuyuki Goto, Prakash Ghosh, Dinesh Mondal

**Affiliations:** 1 Infectious Disease Research Institute, 1616 Eastlake Ave E, Suite 400, Seattle, WA 98102, USA; 2 Department of Animal Resource Sciences, Graduate School of Agricultural and Life Sciences, University of Tokyo, 1-1-1 Yayoi, Bungkyo-ku, Tokyo 113-8657, Japan; 3 68 Shaheed Tajuddin Ahmed Saranai, Mokakhali, Dhaka-1212, Bangladesh

**Keywords:** *Leishmania*, protozoa, biomarkers, diagnosis

## Abstract

Visceral leishmaniasis (VL) in the Old World is caused by infection with *Leishmania donovani*. Although the numbers of new reported cases of VL in Africa have been relatively stable for several years, the low numbers currently reported on the Indian subcontinent suggest a positive impact of new treatments and intervention strategies. In both regions, however, VL relapse and post-kala-azar dermal leishmaniasis (PKDL) maintain infectious reservoirs and therefore present a threat to control programs. In this review, we outline the evolving appreciation of PKDL as an impactful disease in its own right and discuss the various diagnostic methods that can be applied for the detection and characterization of PKDL cases. We also highlight the data that indicate the potential, and likely contribution, of PKDL cases to ongoing transmission of *L. donovani*.

## Teaser

A minireview article emphasizing the importance of the post kala-azar dermal leishmaniasis sequelae to visceral leishmaniasis, with particular attention given to the social and diagnostic limitations that are currently experienced, along with the tools that are emerging that could change the situation for the better.

## INTRODUCTION

**Figure 1.**
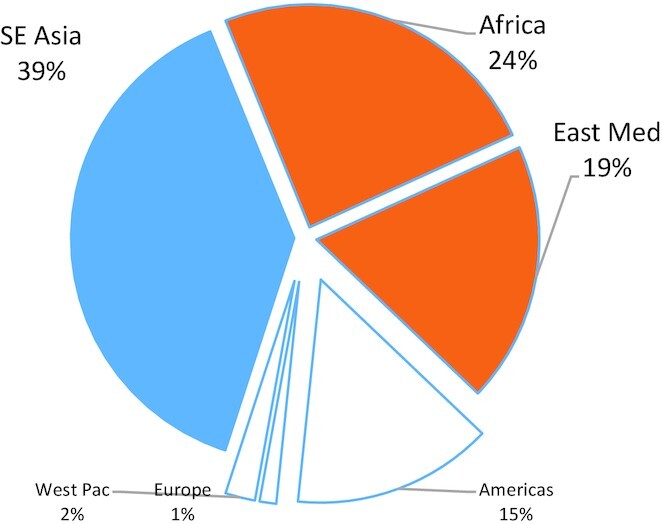
Worldwide distribution of VL in 2015. The WHO regional distribution of VL cases reported to WHO in 2015 is plotted (WHO 2017). Regions that report autochthonous PKDL are shown in color, while those that do not are shown in white. ^1^By WHO reporting structure, Somalia and Sudan are considered to be within the Eastern Mediterranean Region. Three eco-epidemiological hotspots emerge in the region of East Africa (Ethiopia, Kenya, Somalia, South Sudan, Sudan and Uganda)^1^, the Indian subcontinent (Bangladesh, India and Nepal) and Brazil.

Visceral leishmaniasis (VL; also known as kala-azar) results from infection with either *Leishmania donovani* or *Leishmania infantum*. VL presents with generalized symptoms such as splenomegaly, irregular fever, anemia or pancytopenia, weight loss and weakness that develops gradually over a period of weeks or even months that eventually prompts treatment. The 60th World Health Assembly in 2007 adopted Resolution WHA60.13 that requested WHO to raise awareness of the global burden of leishmaniasis and to monitor progress under its control. In 2015, when defined as at least one autochthonous case reported and the demonstration of the entire cycle of transmission within that country, 75 (38%) of the 200 countries or territories reporting to WHO could be considered as endemic for VL. In absolute numbers, 23 804 new VL cases were reported to WHO, with Brazil, India, South Sudan and Sudan each reporting >2000 new cases. Together with Ethiopia, Kenya and Somalia, 90% of VL cases reported worldwide were focused in these countries (WHO 2017). Reporting identifies three eco-epidemiological hotspots: the region of East Africa (Ethiopia, Kenya, Somalia, South Sudan, Sudan, Uganda), which reported 9602 (40%) cases, the Indian subcontinent (Bangladesh, India and Nepal), which reported 9249 (39%) cases, and Brazil, which reported 3336 (14%) cases (Fig. 1).

The Kala-azar Elimination Programme (KAEP) for the Indian subcontinent was formalized in 2005 and since then has made great strides toward its goal of eliminating VL from the region. Among other things, KAEP has strived for integrated vector management and surveillance, social mobilization and partnerships, active and passive surveillance to facilitate case detection, early diagnosis and complete case management. Successes have contributed to attainment of the VL elimination target of <1 case per 10 000 individuals in most areas on the Indian subcontinent (WHO 2005; Zijlstra *et al*. 2017). Translational and operational research has made possible less toxic and simpler treatment regimen and VL patients can now be treated as outpatients without the need for the long-term hospital stays and daily infusions that were previously required. Miltefosine was included as a first-line treatment for VL in 2005 despite its 28-day treatment course, relatively high cost and poor compliance with treatment guidelines (specifically in India where adherence is complicated due to procurement over the counter at the expense of the patient) (Sundar and Murray 2005; Sunyoto *et al*. 2018). AmBisome (liposomal amphotericin B, LAmB; donated by Gilead Sciences to WHO from 2012 to 2017 and recently extended for a further 5-year period) has altered the treatment of VL in Africa and on the Indian subcontinent. Based on active case detection following a diagnostic algorithm, these new treatments have prompted the rapid reduction of VL burden.

As control programs transition to a consolidation phase, vigilance is still required. Cautious interpretation of the impact of various control strategies is also prudent because it has long been observed that VL epidemics occur in cycles, with the most recent cycles peaking in the late 1970s, early 1990s and mid-2000s. Furthermore, even when treatment has led to parasitological ‘cure’ in a VL patient, a substantial subset subsequently develop sequelae that are typically associated with the re-emergence of parasites. Given that sustained elimination of VL requires the disruption of *L. donovani* transmission by the removal of reservoirs, understanding and developing strategies to minimize these sequelae are critical for the success of control programs.

## VL TREATMENT FAILURE AND RELAPSE

In India, miltefosine became a first-line treatment for VL following a phase III trial in Bihar that showed a final cure rate of 94% (Sundar *et al*. 2002). The treatment failure rate has subsequently increased within the last decade of use in South Asia to 10% and this has been accompanied by increased risk of relapse, from 3% to 6.8% (Sundar *et al*. 2012). Treatment failure occurred due to confirmed relapse, not reinfection, in 10.8% at the 6-month and 20% at the 12-month reporting periods of a 120 VL patient cohort, with relapse being more common in children younger than 12 years (Rijal *et al*. 2013). More recently, miltefosine-resistant parasites have been isolated from two Indian VL patients (Srivastava *et al*. 2017). These observations indicate a gradual, and likely irreversible, decline in the efficacy of miltefosine (Pijpers *et al*. 2019). In contrast to the still elongated miltefosine regimen, treatment with LAmB is simplified in that it requires infusion for 3 hours followed by a few hours of observation (Balasegaram *et al*. 2012). Relapses with LAmB occur, on average, 9.6 months after treatment and are more common between 6 and 12 months (0.3% relapsed at 6 months, 3.7% relapsed at 12 months) (Burza *et al*. 2014).

In Eastern Africa, a 30-day sodium stibogluconate (SSG) regimen has been the conventional VL treatment for several decades. Paromomycin (PM; 15 mg/kg/day for 21 days) was tested as a monotherapy, but in contrast to the 94.6% cure rate achieved in India (Sundar *et al*. 2007), the overall efficacy dropped to only 63.8% across African sites. Wide variance in efficacy was observed across study areas, ranging from a high of 96.6% in southern Ethiopia to the lowest levels of only 14.3% and 46.7% observed at sites in Sudan (Hailu *et al*. 2010). A trial of LAmB in Sudan (Gedaref State), and northern and southern Ethiopia (Gondar and Arba Minch, respectively), was terminated due to low efficacy (Khalil *et al*. 2014). Definitive cure rates were 85% in a LAmB multiple dose arm (total dose of 21 mg/kg), reducing to 58% in a single dose 10 mg/kg arm and reducing further to 40% in a single dose 7.5 mg/kg arm. As was observed with PM, inter-regional differences were reported, with southern Ethiopia again presenting the highest cure rate (100% in patients treated with multiple doses or a single dose of 10 mg/kg), whereas Sudan and northern Ethiopia had similarly low efficacy rates (76% and 71% for multiple doses and 39% and 33% for the single 10 mg/kg dose, respectively).

Taken together the data indicate that, although VL treatment options have expanded and regimen has been simplified in recent years, the risk of failure is still present and, worryingly, seems to be increasing.

## POST-KALA-AZAR DERMAL LEISHMANIASIS (PKDL)

**Figure 2.**
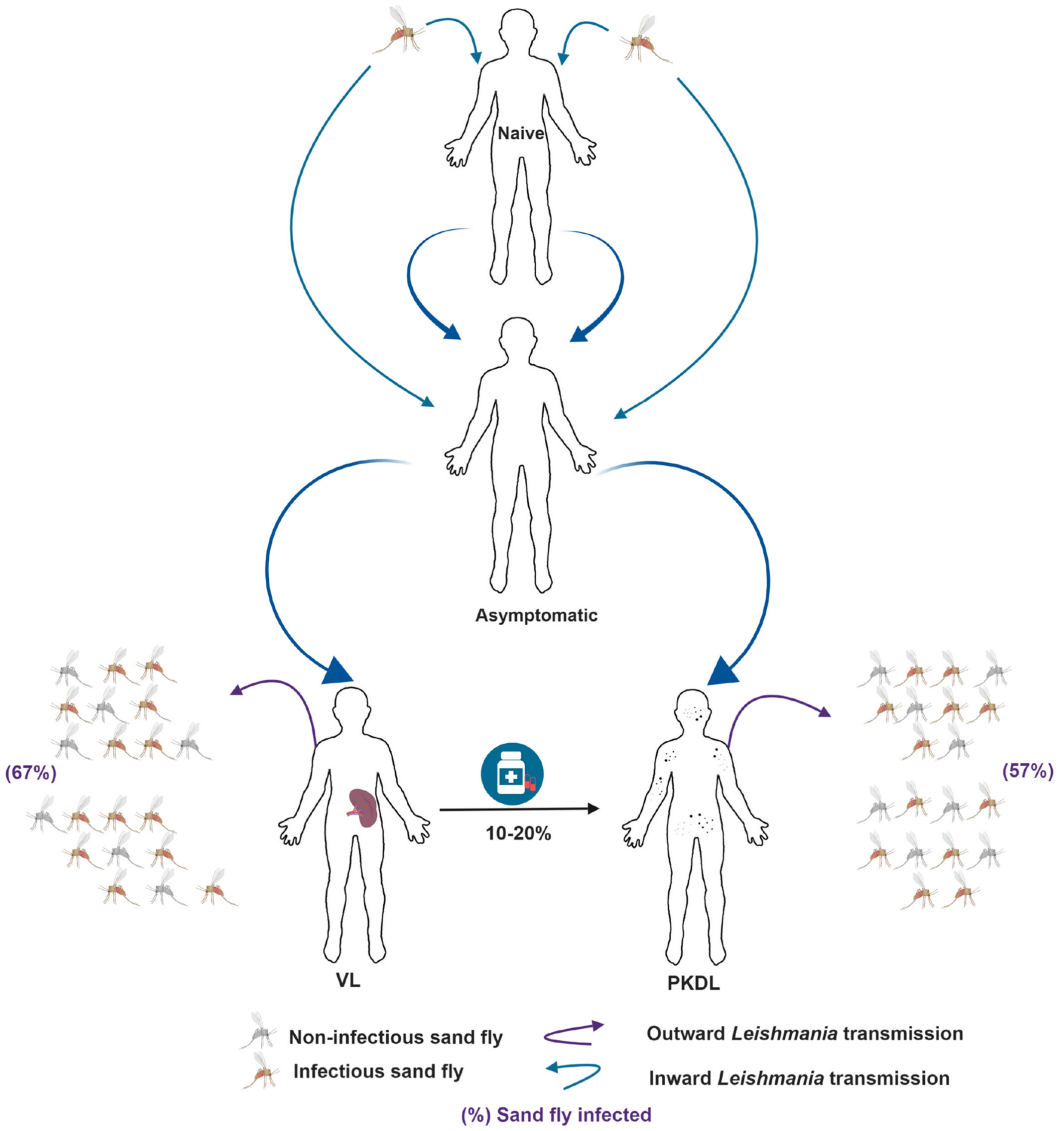
Transmission cycle of Leishmania donovani parasite in anthroponotic VL complications and magnitude of their infectiouness to vector. A systematic depictation of transmission of the parasite to naïve individuals through biting of infectious sand fly; pathogenic progression of the disease from the asymptomatic state; the major organ/ tissue localization of L. donovani in diseased individuals; the parasite uptake (in percent) of sand flies investigated through xenodiagnosis; and the relative proportions of PKDL presentations in L. donovani-endemic regions. PKDL presents most commonly as a sequelae in treated VL patients but can, in a minority, be a primary manifestation of L. donovani infection. The uptake and transmission of L. donovani by sand flies can occur during blood meals on VL and PKDL patients, with asymptomatic infected individuals also likely contributing.

Even after apparently successful treatment, PKDL can occur as a consequence of sustained *L. donovani* infection. Accordingly, peaks in VL incidence have often been followed by peaks in PKDL reporting (Islam *et al*. 2013). PKDL is much more common among treated VL patients than relapse, with case numbers and incidence rates varying between *Leishmania*-endemic regions. The highest rates of 50–60% of all treated VL patients are reported in Sudan, ranging down to rates of 5–20% in India and Bangladesh (Mondal *et al*. 2010; Rahman *et al*. 2010) (Fig. 2). Intriguingly, in some cases PKDL occurs as the primary manifestation of *L. donovani* infection, with an estimated 15–20% of PKDL cases developing in individuals without a recorded history of VL (Ramesh and Mukherjee 1995). Unlike VL, PKDL is not fatal if it remains untreated. In Sudan, it is widely believed that most PKDL lesions gradually resolve on their own accord even in the absence of treatment.

**Figure 3.**
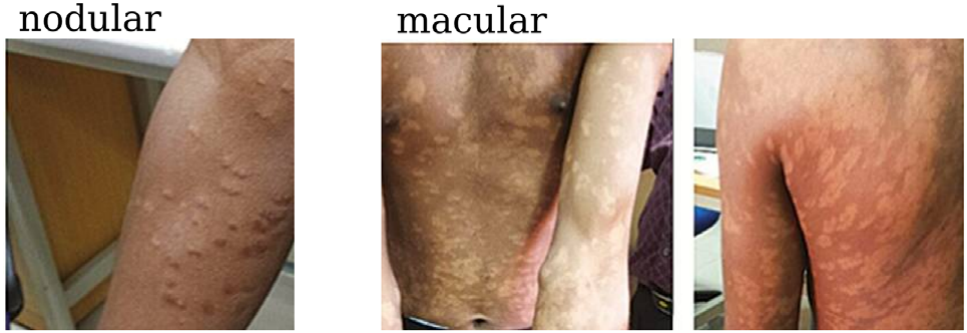
Various presentations of PKDL. PKDL is collectively the manifestation of lesions or hypo-pigmented skin rashes and is characterized by papular, macular and/or nodular lesions over the body. Shown are nodular lesions on forearm and extensive macular lesions on forearms, abdomen and back. Images are the authors' own.

PKDL does not have a singular presentation but is rather the collective manifestation of lesions or hypo-pigmented skin rashes that can be characterized by papular, macular and/or nodular lesions. Lesions often emerge in patients after successful treatment for VL and typically manifest within weeks to a few months after VL treatment (Zijlstra *et al*. 2003). These can occur all over the body, but are mainly found on the face, trunk, legs, arms and genitals (WHO 2005) (Fig. 3). In Sudan, with an incidence rate of 51% nodular or papular lesions are more prevalent in PKDL patients than the incidence for the macular, papular, nodular and polymorphic lesions that are observed at 23%, 17% and 9%, respectively (Ganguly *et al*. 2010). In Bangladesh, the most prevalent form of PKDL is macular whereas in India the distribution of macular and other polymorphic nodular lesions are 23% and 45%, respectively (Ganguly *et al*. 2010; Mondal *et al*. 2010). Of note, however, a recent active surveillance program in West Bengal revealed important differences with PKDL cases who sought treatment in government hospitals with the detection of a higher proportion of macular cases indicating that this may not actually be such an uncommon presentation in that state (Sengupta *et al*. 2019).

Although PKDL is widely regarded as being of no particular concern to the patient as it does not typically interfere with the routine activities of affected individuals, PKDL patients with severe rashes can suffer physical discomfort and are often victims of social stigma (Mukhopadhyay *et al*. 2014). The negative impacts that PKDL has on its sufferers are only now beginning to be accurately documented. In an important, recent controlled assessment of the health-related quality of life in a total of 92 PKDL patient cases and 96 healthy participants who completed the Dermatology Life Quality Index (DLQI) and SF-36 evaluation of general health questionnaires, it was revealed that PKDL patients actually experienced a considerable negative impact on their quality of life (Pal *et al*. 2017). In context, although lower than chronic skin disorders such as burns (17.7) and psoriasis (12.8), with a mean DLQI score of 11.41 the impact of PKDL was worse than that recorded for cutaneous leishmaniasis (5.87), acne vulgaris (8.18) and alopecia (8.3). More than half of the PKDL patients reported a very large negative effect, a rate much higher than the 15% of cutaneous leishmaniasis patients who also experienced this (Vares *et al*. 2013). By SF-36 questionnaire, relative to the healthy control group, PKDL patients showed significant decline in various aspects, including mental health, social functioning, body pain and general health. PKDL patients also attained lower scores for the emotional, mental health and social functioning components of the questionnaire. This probably indicates that psychological and social factors were interfering with the quality of life of PKDL patients involved in that study, with the highest impact on symptoms and feelings and the lowest impact observed in personal relationships. Not surprisingly, however, PKDL patients younger than 20 years had the lower quality of life. In another study, the most affected area of stigmatization was the shame and embarrassment experienced by 68.3% (82 of 120) of the PKDL patients, with one of the most detrimental complications being difficulty in arranging marriage (24.2%) (Garapati *et al*. 2018). These revelations are potentially of enhanced importance given that active long-term (2002–2010) surveillance to detect PKDL cases in Bangladesh found a median patient age of only 12 years and that VL patients younger than 15 years were the most likely to develop PKDL (Islam *et al*. 2013).

## DIAGNOSING PKDL

Stigma likely compounds, and is in turn compounded by, the inappropriate health-seeking behavior that is common among PKDL patients. Simultaneously, lack of knowledge may also increase the length of delay in receiving treatment. The choices of first health care provider sought and distance to primary health care center are significant contributors to these delays (Garapati *et al*. 2018). The median patient delay (time between appearance of symptoms and first medical consultation) has been reported as 285 days (ranging from 15 days to 15 years) with only 71% of patients having approached a health care provider within 1 year of skin lesion development. In addition to these deficiencies increasing the duration and severity of disease, they may also prolong the transmissibility of *L. donovani*.

In the event that a PKDL patient does seek treatment, diagnosis is often complicated by varied manifestations and their symptomatic resemblance to other diseases such as vitiligo, leprosy, secondary syphilis and sarcoidosis. There is no standard guideline for PKDL diagnosis and treatment, and the diagnostic methods available across each PKDL presentation are typically limited to clinical examination and/or standard confirmation techniques such as parasitological examination by slit-skin smear, microscopy and culture. The sensitivity of amastigote detection in slit-skin or skin biopsy smear under microscopy is far from ideal, however, with macular PKDL cases being particularly problematic (ranging from 3.6% to 41.6%) (Mondal *et al*. 2010; Verma *et al*. 2013). Thus, reliable diagnostic and prognostic assay of PKDL are still required.

Several serological and immunological methods have been developed to overcome the limitations of microscopy-based diagnostic methods (Chappuis *et al*. 2007). Despite their use for primary VL diagnoses, rK39 rapid diagnostic tests (RDTs) are used only as an auxiliary test because the persistence of anti-rK39 antibodies in VL patients long after treatment completion precludes their use as the sole confirmatory diagnostic tool for PKDL (Chappuis *et al*. 2006). Interestingly, relative to cured VL patients, *L. donovani* antigen-specific IgG1 levels have been reported as being significantly elevated in relapsed. Analyses of paired samples from Indian VL patients revealed that although IgG1 levels had not decreased significantly at day 30 after treatment initiation, they had dramatically decreased after 6 months. Two prototype lateral flow immunochromatographic RDTs were developed to detect IgG1 levels following VL treatment and provided a clear discrimination of groups: >80% of the relapsed VL patients were IgG1 positive whereas at least 80% of the cured VL patients were IgG1 negative. Thus, whereas no IgG1 or low levels were detected in cured VL patients 6 months after treatment, elevated levels of specific IgG1 were detected in, and associated with, patients displaying treatment failure and relapse (Bhattacharyya *et al*. 2014). More recently, although the predictive value of IgG1 for development of PKDL was not assessed, it was reported that 43 of the 45 (95.6%) Indian PKDL samples were positive in the IgG1 ELISA (Marlais *et al*. 2018). We demonstrated that, unlike antibodies against rK39 and rK28, antibodies against some protein antigens do decline in response to treatment (Vallur *et al*. 2015). The prediction of those patients most likely to succumb to PKDL may therefore be possible based upon particular antigen-specific antibody levels, although the use of these responses to detect/diagnose PKDL would likely be constrained to patients for whom earlier information (i.e. levels at time of VL diagnosis or end of treatment) was available.

While the rate of decay in circulating antibody responses is dictated by the half-life of IgG isotypes (∼21 days), parasite clearance (or persistence) may be more immediately reflected through the measurement of parasite antigens. Indeed, we developed two highly specific capture ELISA based on the detection of native *L. donovani* proteins in urine samples. With the exception of some Sudanese samples, the *Leishmania* Antigen ELISA and *Leishmania* Antigen Detect ELISA were comparable in performance. When responses after treatment initiation were monitored by the *Leishmania* Antigen Detect ELISA, the proportion of positive responses fell from 95% at day 0, to 21% by day 30, and then to all samples being negative by day 180, corresponding with clinical cure (Vallur *et al*. 2015). Refinement to using particular defined antigens may also be instructive and potentially more amenable to large-scale production. For example, glycoproteins (9-OAcSA) have been reported as promising biomarkers of Indian VL patients but it was only recently that their status in PKDL patients was investigated (Jaiswal *et al*. 2018). The glycozylation profiles of isolated immune complexes from PKDL patient sera were analyzed through gradient SDS gel electrophoresis followed by Periodic acid–Schiff (PAS) silver double staining, revealing the presence of several glycan-rich proteins that appeared specific to PKDL. To further characterize antigens present in the circulating immune complexes, glycozylation was demonstrated and then excellent diagnostic performance was revealed in a colorimetric Glyco CIC assay (an AUC value of 0.99). Good prognostic utility was revealed by longitudinal monitoring of 18 PKDL patients. A recently described *Leishmania* 40S ribosomal protein S12 sandwich ELISA also appears to warrant further testing having provided proof-of-concept that it can detect and quantify parasites in peripheral blood mononuclear cell lysates prepared from healthy controls, VL patients and PKDL patients (Zhang *et al*. 2018). The ELISA could detect as few as 60 *L*.*donovani* parasites spiked into cells from healthy donors and capture the target antigen from blood of 68% of VL patients and PKDL patients while providing an estimation of parasitemia ranging from 15 to 80 amastigotes per ml of blood. Some refinement and/or combination of the described *Leishmania* antigen-detecting assays could potentially yield more sensitive detection tests.

From a practical perspective, the collection of easier-to-obtain analytes could enhance usage and therefore make monitoring of VL patients for the emergence of PKDL more common place. It remains to be seen if the non-invasive *Leishmania* Antigen Detect ELISA method developed to detect parasite antigens in urine during acute infection and monitor its clearance upon cure can be used to detect the emergence of PKDL. Of note, however, sweat and urine have been used in rK39 RDT and imply that antibody and antigen capture assays can be adapted to additional analytes to blood/serum. One study in India found that 96.6% of the 58 VL patients evaluated had detectable anti-rK39 antibodies in their sweat, the same detection rate as was attained with blood. Moreover, evaluation of both sweat and blood from 50 PKDL patients found that all had anti-rK39 antibodies in each biological fluid (Topno *et al*. 2018).

Additional direct detection strategies have emerged in the form of various recently described molecular methods (Salam *et al*. 2010; Hossain *et al*. 2017). A species-specific polymerase chain reaction (PCR) using skin slits was compared against *L. donovani* demonstration (achieved by either smear or culture) and rK39 RDT for PKDL case detection in an active surveillance study (Ganguly *et al*. 2015). Among the diagnostic methods used, PCR was the most sensitive (88.46%) for case confirmation, with a prevalence rate of 27.5% among the rK39 RDT positive individuals evaluated. Although conventional PCR using skin specimen yields an encouragingly high sensitivity for PKDL diagnosis (Osman *et al*. 1998), the relatively longer work flow and lack of parasite quantification (an imperative if patient monitoring is desired) limit its potential as a routine diagnostic tool (Salotra *et al*. 2001; Sreenivas *et al*. 2004; Antinori *et al*. 2007; Salam *et al*. 2010). As alternatives, *Leishmania*-specific nested PCR and both single and multiplex real-time PCR methods that retain significant sensitivity and specificity have been developed for the diagnosis of PKDL (Wortmann *et al*. 2005; Verma *et al*. 2010; Weirather *et al*. 2011; Pita-Pereira *et al*. 2012; Sudarshan *et al*. 2014). Nested PCR (91.9% positive samples) compared favorably when evaluated against imprint smear microscopy (70.9% positive samples) for 62 PKDL samples, and an even greater performance gap for these assays was observed when only macular lesions were considered (87.5% by nested PCR compared to 41.6% by imprint smear microscopy) (Verma *et al*. 2013). We reported a preliminary sensitivity of 85% in diagnosing PKDL with skin biopsy specimen by a TaqMan-based real-time PCR method with a limited number of archived samples (Hossain *et al*. 2017), and then more recently validated the assay on a larger scale by comparing its diagnostic efficacy against conventional microscopy (Ghosh *et al*. 2018).

Although there are several potentially useful diagnostic tests for PKDL, the quality of studies evaluating their performance is somewhat constrained and confounded by the poor sensitivity of the reference standard (clinical symptoms rather than confirmation by microscopy) (Adams, Versteeg and Leeflang 2013). This also creates the dilemma that a test that has improved performance will appear to generate false positive results and an apparent decline in specificity. Irrespective of this technical issue, a highly sensitive diagnostic tool remains a highly desirable tool for resolving the problem of PKDL and the validation and implementation of additional tools, including qPCR, appears an imperative for prognosis, diagnosis and monitoring of PKDL.

## PKDL AND SUSTAINED *L. DONOVANI* TRANSMISSION

A previous investigation suggested that PKDL patients comprised the interepidemic reservoir that was also capable of introducing transmission into new areas (Addy and Nandy 1992). Unlike *L. infantum* for which dogs serve as the primary domestic reservoir of parasites and ownership of infected dogs increases the risk of human VL, *L. donovani* appears to be constrained to humans. Assuming treatment access, VL cases can subsequently be tracked using facility-based data but nonetheless PKDL cases often remain undetected (Mondal *et al*. 2010; Molina *et al*. 2017). It is noteworthy that a recent study in West Bengal revealed that many PKDL cases would remain undiagnosed without active surveys (Ganguly *et al*. 2015). Furthermore, even after treatment completion, in the majority of the PKDL cases skin lesions persist before disappearance 6–12 months after the treatment period. Thus, in addition to their own personal impact, PKDL cases are implicated as important reservoirs for *L. donovani* and a likely source of ongoing VL incidence (Zijlstra *et al*. 2017). By extension, elimination, or at least control, of PKDL is critical to achieve the goal of VL elimination.

Xenodiagnosis, i.e. the demonstration of infection in the sand fly subsequent to feeding on the suspected host, is the only means to definitively demonstrate transmission potential. It is almost 100 years since Indian VL patients were first shown to be infectious to sand flies (Christophers, Shortt and Barraud 1924; Knowles, Napier and Smith 1924; Shortt, Barraud and Craighead 1926). Further experiments shortly thereafter revealed that a patient with nodular PKDL lesions was similarly infectious to *Phlebotomusargentipes* (Shortt and Swaminat 1928). By examining longer incubation or repetitive feeding on the same PKDL patient, it was suggested that extended exposures increased the infectious yield (Christophers, Shortt and Barraud 1926). Both nodular and macular PKDL patients could infect sand flies (Napier *et al*. 1933). Advances in parasite detection and enumeration methods have allowed finer analyses. In a preliminary xenodiagnosis study from three patients with either maculopapular or nodular PKDL, and for whom loads were assessed as 1428, 21 621 and 63 058 parasites per microgram of skin biopsy material, we demonstrated that each had the ability to infect feeding sand flies (Molina *et al*. 2017). In a more recent study, we found that 57.4% PKDL patients (27 of 47 evaluated) yielded positive xenodiagnoses and that a tentative transmission threshold could be postulated: PKDL patients with positive xenodiagnosis had median skin parasite loads >10-fold higher than the loads measured in those with negative results (Mondal *et al*. 2019). Parasite load and positive skin microscopy were significantly associated with positive xenodiagnoses. Relative to VL patients (of whom 66.6% transmitted), nodular PKDL patients (86%) were more likely and macular PKDL patients (35%) were less likely to support parasite transmission. Strong evidence that PKDL cases can sustain *L. donovani* transmission to sand flies is therefore being generated, highlighting the necessity to consider the role of PKDL in sustained control of VL.

## SUMMARY

At least on the Indian subcontinent, the reported new VL cases are currently low, suggesting a positive impact of new treatments and intervention strategies. Sustaining these reduced numbers and continuing the push to elimination require continued vigilance. Untreated PKDL patients likely serve as a persistent reservoir of *L. donovani* and can contribute to maintenance of the transmission cycle. At present, however, PKDL detection and treatment are important missing, or underappreciated, components of the overall VL elimination program. Various diagnostic tools are emerging that could address this gap if they progress from research to implementation.

## FUNDING

The authors are funded by The Global Health Innovative Technology Fund (G2018–111). Leishmania research at IDRI has also been funded by grants from CRDF Global (#62966), National Institute of Allergy and Infectious Diseases of the National Institutes of Health under Award Number R01AI025038 and the Bill and Melinda Gates Foundation (#631 and #39129). Leishmania research at icddr,b has also been supported by the Thrasher Research Fund (Award Number: 11921), with PG supported by a EDCTP-TDR Clinical Research Development Fellowship 2018. Leishmania research at The University of Tokyo has also been supported by Japanese Society for the Promotion of Science grant (#18H02649), Japan Agency for Medical Research and Development grant for Translational Research Network Program grant Seed A and Japan Agency for Medical Research and Development grant for U.S.–Japan Cooperative Medical Sciences Program.
